# Anaplastic lymphoma kinase-positive inflammatory myofibroblastic tumor of the breast: a case report and review of the literature

**DOI:** 10.1186/s40792-023-01732-6

**Published:** 2023-09-01

**Authors:** Yasutaka Kawakita, Keisei Anan, Kanako Kurata, Kenichiro Koga, Michiyo Saimura, Sadafumi Tamiya, Kazuyoshi Nishihara, Shoshu Mitsuyama, Toru Nakano

**Affiliations:** 1 Department of Surgery, Kitakyushu Municipal Medical Center, 2-1-1 Bashaku Kokurakita-Ku, Kitakyushu, Fukuoka 802-0077 Japan; 2 Department of Pathology, Kitakyushu Municipal Medical Center, 2-1-1 Bashaku Kokurakita-Ku, Kitakyushu, Fukuoka 802-0077 Japan; 3 Department of Surgery 1, School of Medicine, University of Occupational and Environmental Health, 1-1 Iseigaoka Yahatanishi-Ku, Kitakyushu, Fukuoka 807-8555 Japan

**Keywords:** Inflammatory myofibroblastic tumor (IMT), Anaplastic lymphoma kinase (ALK), Spindle cell tumors

## Abstract

**Background:**

Few reports of inflammatory myofibroblastic tumor (IMT) of the breast have been published worldwide. Furthermore, primary anaplastic lymphoma kinase (ALK)-positive IMT of the breast is extremely rare. To date, only six patients with ALK-positive IMT have been reported in the literature.

**Case presentation:**

A 52-year-old woman underwent a medical examination, and a left breast mass was detected. She did not feel a mass in her chest. Mammography showed a focal asymmetric density at the lower outer portion of the left breast. Breast ultrasonography showed a 1.2-cm hypoechoic lesion with relatively clear boundaries and poor blood flow. Magnetic resonance imaging and computed tomography revealed a solitary heterogeneous mass in the left breast. Pathologic examination revealed a fibrosing lesion with proliferation of fibroblastic cells arranged in a storiform pattern and admixed inflammatory cells. Immunohistochemical examination showed that the tumor cells were positive for ALK. Under the preoperative diagnosis of IMT, we performed partial mastectomy with adequate margins. The postoperative diagnosis was pathologically confirmed as IMT. Immunohistochemical staining also showed overexpression of ALK-1 in the tumor. The patient had a good clinical course for 24 months postoperatively, without recurrence or metastasis.

**Conclusions:**

IMT of the breast shows nonspecific imaging findings, making preoperative diagnosis difficult. Nevertheless, IMT has the characteristics of low-grade neoplasms with recurrence, invasion, and metastatic potential. Our report emphasizes the importance of determining a treatment plan as soon as possible based on an accurate diagnosis to improve the prognosis of this disease.

## Background

Inflammatory myofibroblastic tumor (IMT) is composed of myofibroblastic and fibroblastic spindle cells accompanied by admixed inflammatory cells, including lymphocytes, plasma cells, and eosinophils. IMT is a subcategory of inflammatory pseudotumor. In the 2020 World Health Organization classification of tumors of soft tissue and bone, IMT is classified as a low-grade neoplasm characterized by recurrence, invasion, and metastatic potential; however, its detailed clinical course has not yet been elucidated [1]. IMT occurs at a wide range of ages, especially in children and younger adolescents, and it may occur in any soft tissue or visceral location. For example, there are many reports of IMT in the lungs, mesentery, omentum, pelvic organs, mediastinum, larynx, central nervous system, and retroperitoneum [2, 3]. However, primary IMT of the breast is uncommon, and recurrence and metastasis are fairly rare. In addition, preoperative diagnosis of IMT is quite difficult; therefore, the diagnosis is often confirmed only by surgical resection or biopsy according to many reports. In recent years, expression of anaplastic lymphoma kinase (ALK) or the presence of rearrangements associated with the ALK gene have been regarded as important findings [4]. Although the proportion of ALK-1-positive cases varies depending on the site of occurrence, ALK-1 is generally expressed in about 40 to 50% of IMTs [5]. Surgical resection is the only curative treatment at present, but local recurrence or distant metastasis of IMT can occur even after complete resection. Therefore, prompt diagnosis including the ALK status is very important in determining the treatment strategy for IMT.

In this case report, we describe a case of ALK-positive IMT of the breast in a middle-aged woman with no history of chest surgery or trauma. We achieved the diagnosis before surgery by pathological findings and immunohistochemical examination. Herein, we report our case and compare our findings with previously published reviews of the literature from around the world.

## Case presentation

**Fig. 1:**
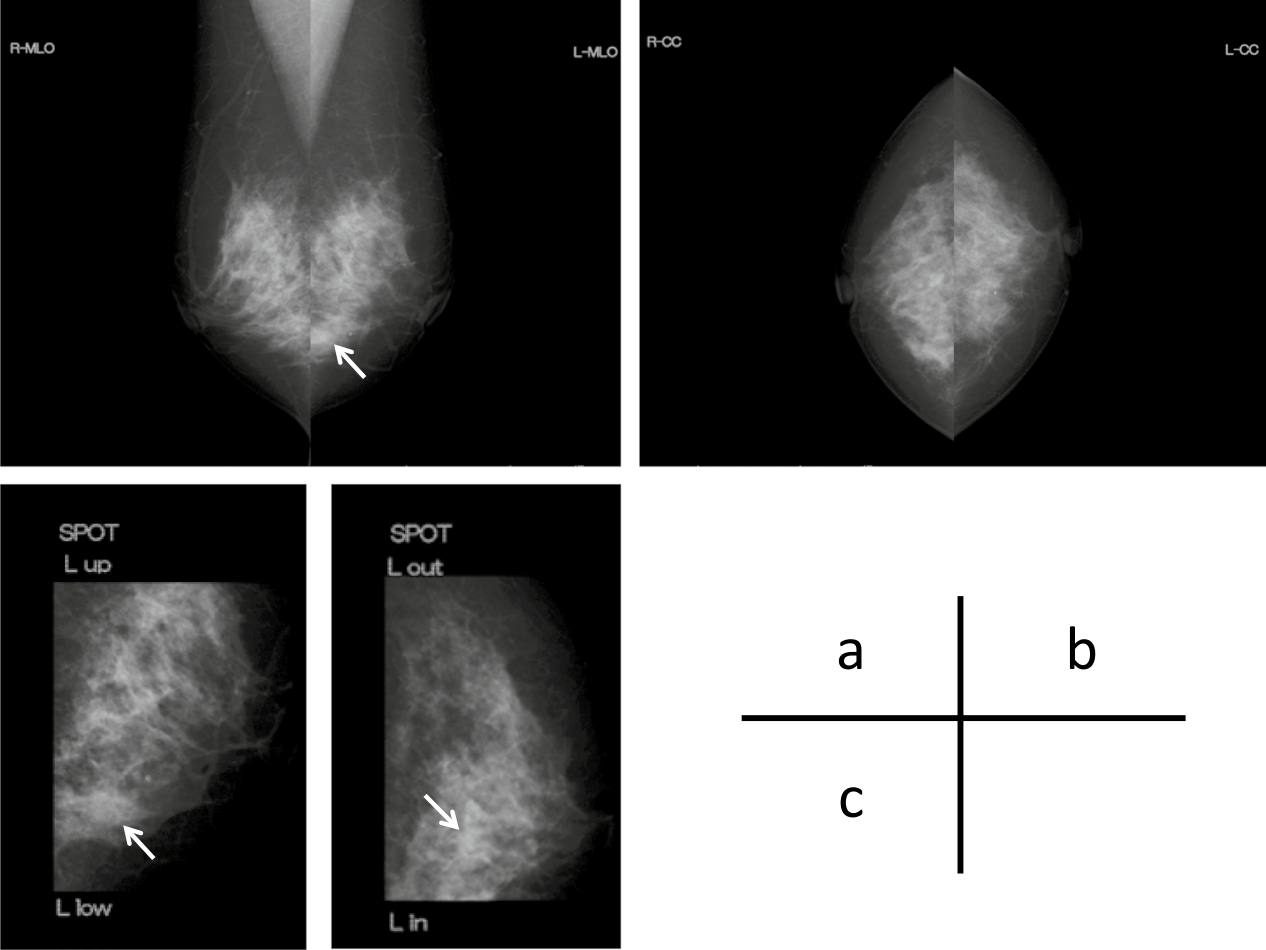
Mammography. **a** Mediolateral oblique view: focal asymmetric density at the lower portion of the left breast. **b** Craniocaudal view: no clear anomalies because of overlapping with the mammary gland. **c** Spot compression view: slight focal asymmetric density at the outer portion

**Fig. 2:**
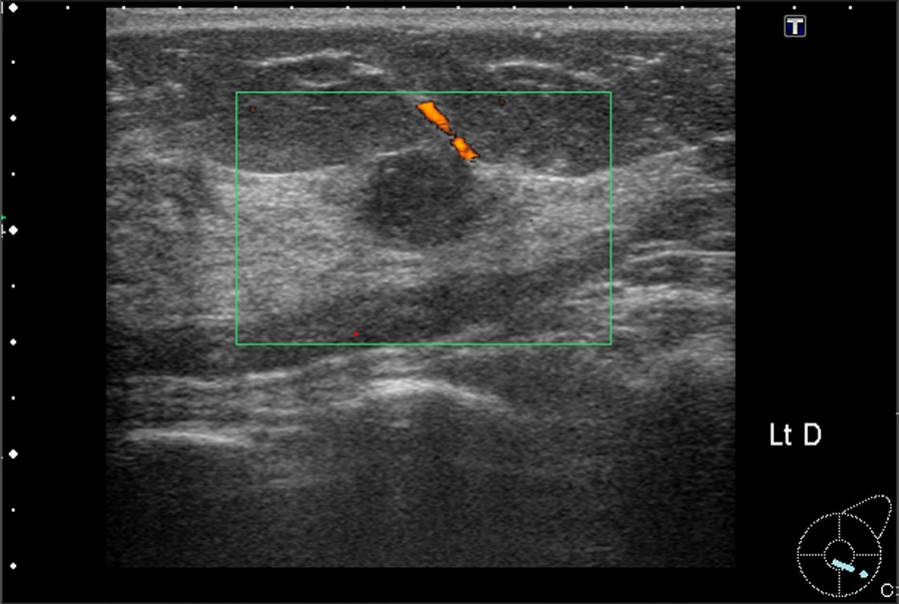
Ultrasonography. A 1.2-cm-diameter relatively smoothly marginated hypoechoic mass with poor internal blood flow

**Fig. 3:**
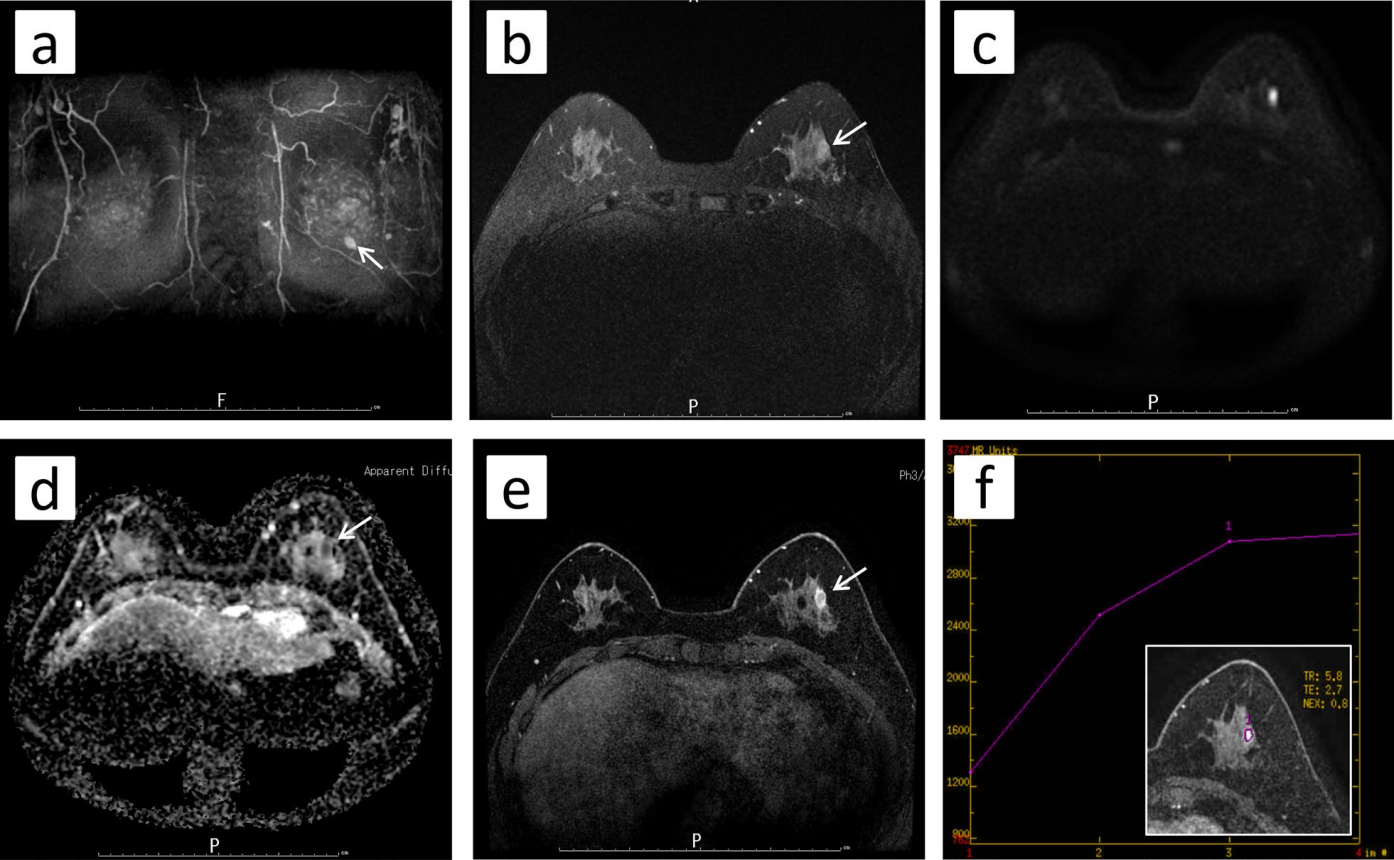
Magnetic resonance imaging. **a** A 1.2-cm-diameter mass at the lower outer portion of the left breast. **b** T2-weighted imaging: Mildly high signal intensity at the periphery and center of the tumor. **c** Diffusion-weighted images: high signal intensity. **d** Apparent diffusion coefficient map: low signal intensity. **e** Dynamic contrast-enhanced magnetic resonance imaging: hyperintensity in the early phase. **f** Time–intensity curve: intermediate enhancement in the initial period and a plateau in the delayed period

**Fig. 4:**
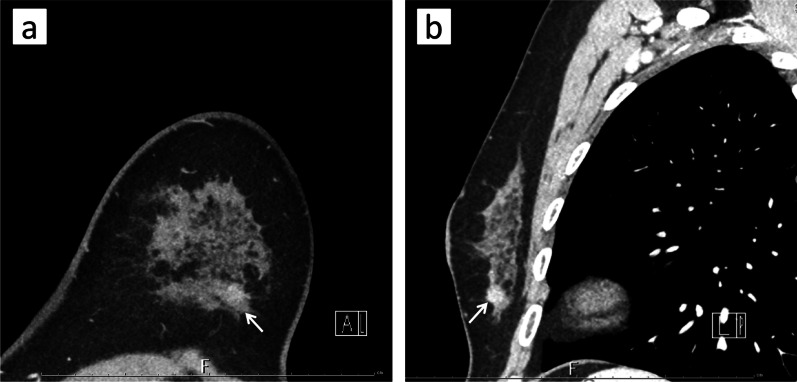
Enhanced computed tomography. Solitary heterogeneously enhanced mass at the outer lower area of the left breast

A 52-year-old woman underwent a medical examination at a local clinic. She did not feel a mass in her breast, but a lump in the left breast was detected by breast ultrasonography. She was referred to our hospital for further investigation. Although she had received laser treatment for cervical dysplasia at the age of 40 years, she had no other clinical history such as cancer or breast trauma. Her family history included breast cancer in a younger sister and gastric cancer in her father. Physical examination revealed no palpable mass in her breast and no swollen axillary lymph nodes. Mammography revealed a focal asymmetric density at the lower outer portion of the left breast (Fig. 1a–c). Breast ultrasonography showed a heterogeneous 1.2-cm hypoechoic lesion with partially cystic changes and relatively clear boundaries. Vascular signals were scant (Fig. 2). Magnetic resonance imaging revealed a mass approximately 1.2 cm in diameter at the lower outer portion of the left breast. T2-weighted imaging showed mildly high signal intensity at the periphery and center of the tumor (Fig. 3a, b). The signal was elevated on diffusion-weighted imaging but low in the apparent diffusion coefficient map (Fig. 3c, d). Dynamic contrast-enhanced magnetic resonance imaging showed hyperintensity in the early phase, but the signal intensity was slightly attenuated in the center of the tumor in the delayed phase (Fig. 3e). A time–intensity curve of the mass showed intermediate enhancement in the initial period and a plateau in the delayed period (Fig. 3f). Enhanced chest computed tomography also detected a solitary mass with heterogeneous rim enhancement at the outer lower area of the left breast (Fig. 4). No lymph node swelling or distant metastasis was present.

**Fig. 5:**
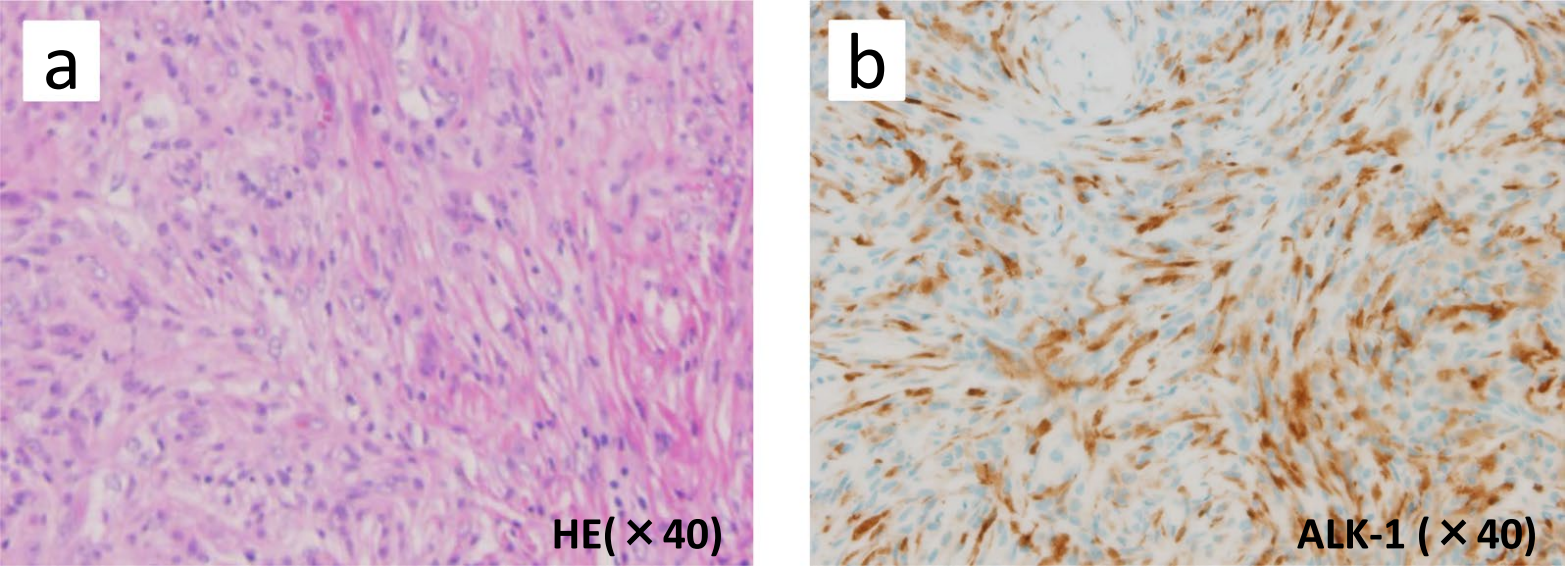
Core needle biopsy. **a** Fibrosing lesion with proliferation of fibroblastic cells arranged in a storiform pattern, chronic inflammatory cells, and histiocytic cells. **b** Immunohistochemical examination: positivity for ALK-1. *HE* hematoxylin–eosin, *ALK-1* anaplastic lymphoma kinase-1

A tumor specimen was obtained by percutaneous biopsy. Core needle biopsy of the left breast mass showed a fibrosing lesion with proliferation of fibroblastic cells arranged in a storiform pattern, chronic inflammatory cells, and histiocytic cells (Fig. 5). No epithelial neoplasm was observed. The spindle cells in the lesion were immunohistochemically positive for ALK.

At the initial examination, the C-reactive protein concentration was slightly increased at 0.16 mg/dL (reference range, 0.00–0.14 mg/dL). The carcinoembryonic antigen and cancer antigen 15-3 concentrations were not increased. The serum concentrations of other indexes were within the reference ranges. The serum interleukin-6 concentration was also within the reference range.

**Fig. 6:**
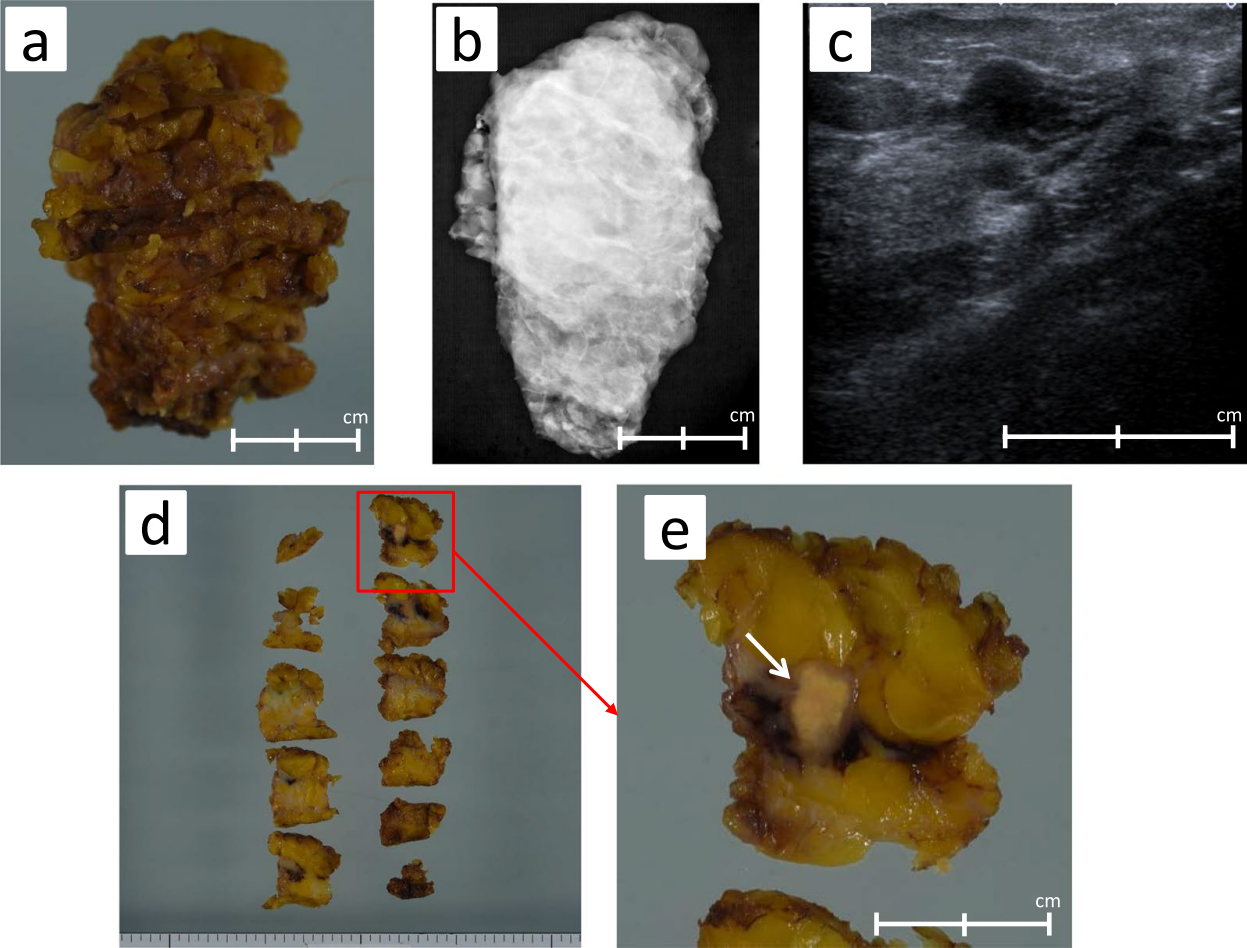
Excised specimen. **a** Partial mastectomy with adequate margins. **b** No significant findings on X-ray photography of the intraoperative specimen. **c** A 1.2-cm-diameter hypoechoic mass with ill-defined margins in the center of the specimen by intraoperative ultrasonography. **d**, **e** The well-defined 1.0- × 0.7-cm tumor was homogeneous and grayish on cut section

**Fig. 7:**
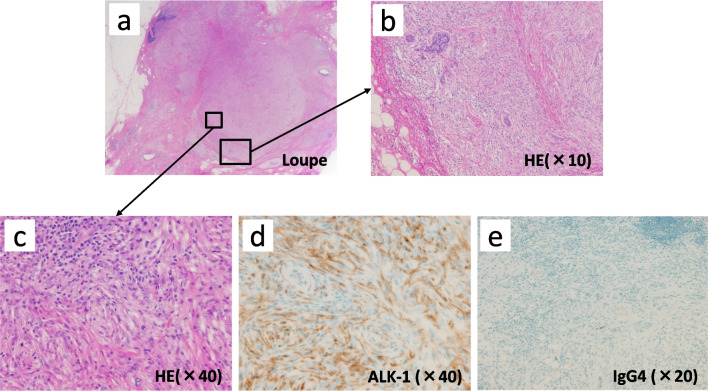
Histopathological examination (HE stain). **a** Ill-defined tumor with slightly fibrotic surrounding mammary glands. **b** Proliferation of fibroblastic cells arranged in a storiform pattern (× 10). **c** Infiltration of chronic inflammatory cells and histiocytic cells with variously sized spindle cells (× 40). **d**, **e** Immunohistochemical examination: diffuse positivity for ALK-1 (× 40) and negativity for IgG4 (× 20). *HE* hematoxylin–eosin, *ALK-1* anaplastic lymphoma kinase-1

Under the preoperative diagnosis of IMT, we performed left partial mastectomy with adequate margins (> 1.0 cm) for a definitive diagnosis. There were no significant findings on intraoperative specimen X-ray photography. However, intraoperative ultrasonography of the excised specimen showed a 1.2-cm hypoechoic mass in the center of the specimen with ill-defined margins (Fig. 6a–c). The specimen showed a well-defined 1.0- × 0.7-cm tumor that appeared homogeneous and gray–white on cut section (Fig. 6d, e). The postoperative pathologic examination revealed a fibrosing lesion with proliferation of fibroblastic cells arranged in a storiform pattern. The surrounding mammary tissue showed fibrocystic change (Fig. 7a, b). Chronic inflammatory cells and histiocytic cells were also observed (Fig. 7c). The surgical margins were clear of tumor cells. Immunohistochemically, the tumor cells were diffusely positive for ALK-1 and negative for IgG4 (Fig. 7d, e). There were no histopathological findings of breast cancer. The final diagnosis was pathologically confirmed as IMT. At the time of this writing, the patient was being followed up and was doing well 24 months postoperatively without recurrence or metastasis.

## Discussion

When we searched PubMed using the keyword “inflammatory myofibroblast tumor of the breast” and excluded the proceedings published from 1988 to 2022, we found 4325 reports of IMT worldwide. Among them, only 35 described IMT arising in the breast; our case is the 36th (Table 1) [6–36]. IMT was first reported in the lungs in 1939 [37]. IMT of the breast was first described by Pettinato et al. [6] in 1988. Approximately 43% of extrapulmonary IMT arises in the mesentery and omentum [38]. ALK-positive primary IMT of the breast is extremely rare. Currently, IMTs are regarded as intermediate neoplasms with potential for local recurrence and rarely metastasis. Metastases of IMT typically occur in the lungs, brain, liver, and bone. Previously published cases of recurrent or metastatic IMT of the breast have involved patients with a broad range of ages and metastatic sites. Local recurrence of IMT has been recorded in about 10 to 25% of cases [5, 30]. Distant metastasis of IMT is extremely rare, occurring in < 5% of cases [26]. A report in Japan described rapid enlargement of a low-grade malignant spindle cell tumor within 1 year [39]. Rare malignant transformation after complete resection has also been reported [27]. In such cases, not only surgery but also chemotherapy or radiotherapy have been used. However, the effect of these treatments is quite limited. We found no large-scale studies on the clinical response to systemic therapy in IMT of the breast; thus, there is limited evidence on the selection of appropriate treatment for patients with IMT of the breast. No consensus has yet been reached; only surgical resection is considered a curative treatment with a good outcome [19, 34]. Gleason and Hornick [40] also reported that the recommended treatment is complete surgical excision and that recurrence rates of up to 25% are experienced. Furthermore, many reports have emphasized the importance of preoperative examination to determine the most appropriate surgical procedure [23, 27, 36].

**Table 1:** Previously published cases of IMT of the breast

Case	Author	Year	Age	Sex	Location	Size (cm)	ALK	Past trauma or surgery	Follow-up	Outcome
1	Pettinato et al. [6]	1988	29	F	Right	4.5	–	–	NED 30 months	–
2	Coffin et al. [7]	1995	13	F	Right	4	–	–	NED 12 months	–
3	Chetty et al. [8]	1997	16	F	Right	2	–	–	NED 12 months	–
4	Chetty et al. [8]	1997	46	F	Right	2	–	–	NED 12 months	–
5	Chetty et al. [8]	1997	18	F	Right	8	–	–	NED 6 months	–
6	Yip et al. [9]	1997	66	F	Left	3	–	None	Recurrence 12 months	Survival
7	Gobbi et al. [10]	1999	86	F	Left	1.5	–	–	–	–
8	Sastre-Garau et al. [11]	2002	64	F	Right	3	Neg	None	NED 33 months	–
9	Haj et al. [12]	2003	31	F	Right	1.8	–	None	–	–
10	Zardawi et al. [13]	2003	79	F	Right	1.5	Neg	–	Recurrences 108 months	Survival
11	Ilvan et al. [14]	2005	60	F	Right	1	Neg	None	NED 85 months	Survival
12	Khanafsar et al. [15]	2005	33	F	Left	2	Neg	None	Recurrence 3 months	Survival
13	Khanafsar et al. [15]	2005	75	F	Left	3	Neg	None	NED 14 months	Survival
14	Khanafsar et al. [15]	2005	47	F	Right	2	Neg	Surgery (right)	NED 12 months	Survival
15	Zen et al. [16]	2005	46	F	Left	1.6	–	–	NED 12 months	Survival
16	Akbulut et al. [17]	2007	38	F	Left	1	Neg	None	NED 12 months	Survival
17	Kim et al. [18]	2009	60	F	Left	1.5	Neg	None	NED 24 months	Survival
18	Park et al. [19]	2009	47	F	Right	3.5	–	None	NED 36 months	Survival
19	Hill et al. [20]	2010	53	F	Right	3.5	Neg	None	–	–
20	Sari et al. [21]	2011	54	F	Left	2.7	–	None	NED 4 months	Survival
21	Vecchio et al. [22]	2011	22	M	Left	7	Neg	Trauma (left)	NED 10 months	Survival
22	Zhou et al. [23]	2013	46	F	Right	1.1	Pos	None	NED 60 months	Survival
23	Li et al. [24]	2013	39	F	Left	4.5	Pos	None	NED 24 months	Survival
24	Zhao et al. [25]	2013	56	F	Right	4	Pos	None	Recurrence and metastasis 3,7,10 months	–
25	Bosse et al. [26]	2014	23	F	Left	2	Pos	None	NED 12 months	Survival
26	Xing et al. [27]	2014	56	F	Right	9	–	None	Recurrence and metastasis 2 months	Death
27	Kovacs et al. [28]	2015	31	F	Left nipple	1.6	Pos	None	NED 60 months	Survival
28	Markopoulos et al. [29]	2015	67	F	Left	1	Neg	None	NED 6 months	Survival
29	Choi et al. [30]	2015	27	F	Right	3	Neg	None	Recurrence and metastasis 24 months	–
30	Talu et al. [31]	2016	38	F	Left	1.5	Neg	None	NED 16 months	Survival
31	Siraj et al. [32]	2017	60	M	Left	15	Neg	None	NED 6 months	Survival
32	Inoue et al. [33]	2018	16	F	Right	2.2	Pos	None	Metastasis 0 months	–
33	Fernandes-Acenero et al. [34]	2018	52	F	Right	5	–	–	NED 8 months	Survival
34	Mao et al. [35]	2018	43	F	Left	1.4	Neg	Surgery (left)	NED 12 months	Survival
35	Wei et al. [36]	2021	50	F	Right	4.5	Neg	Surgery (right)	NED 44 months	Survival
36	Present case	2022	52	F	Left	1.2	Pos	None	NED 18 months	Survival

ー not mentioned or not source, *F* female, *M* male, *Pos* positive, *Neg* negative, *NED* no evidence of disease

In total, 36 cases of primary IMT of the breast, including our case, have been identified to date (Table 1). As shown in Table 1, the oldest patient was an 86-year-old woman and the youngest was a 13-year-old girl. The mean age among the 36 patients was 45.5 years, and the median age was 46.5 years. Twenty patients (55.6%) were aged < 50 years. The size of the tumors ranged from 1 to 15 cm in diameter on imaging examination (average, 3.3 cm; median, 2.1 cm). Six of the 36 patients showed local recurrence during follow-up. The time until recurrence varied from 2 months to 9 years. The total local recurrence rate was 16.7% for all patients. Two of six patients had bilateral breast recurrence. Notably, four metastatic cases have been reported to date, for a metastasis rate of 11.1%. This figure is higher than previously reported. Only two cases of IMT occurred in the male breast. We found no reports on whether or how the prognosis of IMT depends on sex.

Some studies have shown that IMT is likely to be caused by a history of trauma to the breast. Therefore, IMT was initially thought to be associated with post-inflammatory repair processes, such as those associated with trauma, infection, and surgery. Such stimuli activate myofibroblasts to potentially proliferate and form tumors, and IMT was thus recognized as a type of inflammatory pseudotumor. However, as shown Table 1, only four patients had a history of trauma or surgery at the same site where the IMT occurred. It seems that the clinical findings are mostly related to inflammatory conditions, but some of the features of IMT are more similar to the lineage of the tumor pathology than to inflammation. Based on the results of our literature review, the occurrence of IMT is considered to be poorly related to trauma and surgery.

Because of the lack of characteristic imaging findings for IMT of the breast, it is exceedingly difficult to make a definitive diagnosis before surgery. Differential diagnoses that should be considered include breast cancer, myxoma, fibroma, ALK-positive histiocytosis, and IgG4-related inflammatory pseudotumor. In the present case, morphological examination revealed no atypical cells that were consistent with carcinoma or sarcoma. Although the pathological findings were similar to those of an inflammatory pseudotumor-like lesion, the tumor was strongly considered neoplastic rather than reactive because of the presence of spindle cells. Therefore, IMT and IgG4-related inflammatory disease were considered the most likely differential diagnoses. We used immunostaining for IgG4 to rule out diseases other than ALK-positive IMT. Although we did not measure the ratio of IgG4/IgG-positive cells or use immunostaining for α-smooth muscle actin, vimentin, or desmin in this case, these procedures are also reportedly often useful for differential diagnosis. Our patient had no symptoms such as fever, chills, or arthralgia. Interleukin-6 has also been reported to be elevated in patients with IMT, myxoma, and autoimmune diseases, but it was not elevated in our patient. Tumor markers such as carcinoembryonic antigen and cancer antigen 15-3 were also within the reference range. We were able to make a definitive diagnosis by pathological and immunohistochemical examination. Karnak et al. [41] also indicated that the diagnosis was frequently confirmed only by surgical resection or biopsy. In recent years, overexpression of ALK or the presence of rearrangements associated with the ALK gene have been regarded as important findings in diagnosis of IMT. In Table 2, we show the clinical course, prognosis, and treatment in previously published cases of ALK-positive IMT of the breast (Table 2). Only three cases could be preoperatively diagnosed as IMT, and all of these diagnoses were achieved by core needle biopsy. This fact indicates that the preoperative pathological findings, including the ALK status, are important for determining a treatment plan based on an accurate diagnosis. ALK is a receptor tyrosine kinase that has been conserved across species. Recent studies have shown that chromosomes 2 and 9 are abnormal in IMT, and more than half of IMTs exhibit fusion of ALK genes on 2p23 [36]. The ALK-1 expression rate is reportedly about 40% to 50% in IMT arising in the breast [26]. This property also suggests that IMT is a true tumor with a neoplastic nature and not an inflammatory lesion. As shown in Table 1, the rate of ALK-positive IMT was 30.4% among the reported cases. In 2013, Zhou et al. [23] described the first case of ALK-overexpressing IMT of the breast in a 46-year-old woman. The patient was free of recurrence 5 years after the diagnosis.

**Table 2:** Previously published cases of ALK-positive IMT of the breast

Case	Author	Age	Preoperative diagnosis	Biopsy	Treatment	Recurrence/metastasis site	Duration	Follow-up
22	Zhou et al. [23]	46	Malignancy	–	Surgery (excision, axillary LN biopsy)	None	–	NED 60 months
23	Li et al. [24]	39	–	–	Surgery (mastectomy, biopsy)	None	–	NED 24 months
24	Zhao et al. [25]	56	Malignancy	–	Surgery (excision × 2), Radiotherapy	Chest wall, ribs, vessels, left groin	3,7,10 months	Recurrence and metastasis
25	Bosse et al. [26]	23	IMT	CNB	Surgery (partial mastectomy)	None	–	NED 12 months
27	Kovacs et al. [28]	31	IMT	CNB	Surgery (incomplete)	None	–	NED 60 months
32	Inoue et al. [33]	16	No definitive	–	Surgery (partial mastectomy)	Intracranial, lung, pancreas	Same time	Metastasis
36	Present case	52	IMT	CNB	Surgery (partial mastectomy)	None	–	NED 24 months

ー not mentioned or not source, *F* female, *M* male, *NED* no evidence of disease

In general, ALK expression is more common in younger patients with IMT. As shown in Table 2, the mean age among the seven ALK-positive cases was 37.6 years. This age is significantly younger than that of ALK-negative cases (mean of 51 years). ALK positivity is considered to be associated with a less aggressive clinical course. In contrast, ALK-negative IMT is often associated with older age, more aggressive local disease, and occasional metastasis [42]. Many reports have shown that the potential for rapid growth, malignant transformation, local recurrence, and metastasis is frequently correlated with the absence of ALK expression, similar to a high degree of atypia, increased mitotic figures, an elevated Ki-67 proliferative index, and overexpression of oncogenic proteins such as p53 [28, 29]. Choi et al. [30] found a higher degree of nuclear pleomorphism, atypia, and atypical mitoses in patients with ALK-negative IMT. However, whether patients with ALK-positive IMT truly have a more favorable outcome is not known. As indicated in Table 2, two cases of metastatic IMT showed overexpression of ALK. Although the risk of metastasis is reportedly very low, the incidence of recurrence and metastasis remains questionable. Because the role of ALK gene amplification in IMT of the breast is still unclear, further studies are required.

Because the resection margin was adequate in the present case, we did not perform chemotherapy or additional treatment. No reports to date have described the safe resection margin for IMT of the breast. Additionally, whether the safe resection margin for IMT varies by organ remains unclear. Many case reports on IMT regarding various organs have indicated that complete excision with a negative and adequate margin is the preferred treatment and results in the lowest chance of disease recurrence [43, 44]. One report in Japan described recurrence from the liver stump after left hepatectomy for IMT [45]. In a case of IMT of the breast, Bosse et al. [26] reported that they attempted to secure a resection margin of at least 10 mm. IMTs have the characteristics of intermediate-grade neoplasms; therefore, when treating IMT of the breast, it is necessary to secure a resection margin similar to that of breast cancer. However, excessive therapy (total mastectomy, axillary lymph node dissection, or radiotherapy) is unwarranted given the indolent and often benign clinical course in the majority of cases. There seems to be no objection to the importance of securing a negative and sufficient resection margin.

Although IMT is usually cured by resection with a large enough margin, it can sometimes show rapid growth, infiltration, recurrence, and metastasis [25, 39]. Despite their serious complications, ALK inhibitors have shown high response rates in patients with ALK-positive lung cancer worldwide. In recent years, many reports have described the use of ALK inhibitors for ALK-positive IMT. Takeyasu et al. [4] reported seven cases of ALK-rearranged nonlung solid tumors, including IMT, treated with ALK inhibitors. The objective response rate for the initial ALK inhibitor therapy was 85.7% (95% confidence interval, 44–97), including two patients who achieved a complete response. Another report described preoperative treatment using the neoadjuvant ALK inhibitor crizotinib for tumor reduction of locally advanced IMT of the bladder, enabling bladder-preserving surgery without recurrence [46]. The authors concluded that neoadjuvant ALK inhibitor therapy may be effective for large, locally advanced, and difficult-to-resect tumors. In a rare case, complete remission was achieved by using an ALK inhibitor for multiple recurrences of pelvic IMT after complete surgical excision [47]. These reports suggest that ALK inhibitors have significant benefit for ALK-positive solid tumors, including IMT.

Several basket trials of patients with ALK-positive solid tumors are ongoing. Representatives among these are DETERMINE (NCT 05770037), STARTRK-NG (NCT02650401), and STARTRK-2 (NCT02568267). These trials are being conducted at multiple sites worldwide. Patients will be assigned to different baskets according to their tumor type and gene fusion. DETERMINE is part of a trial program examining various anti-cancer drugs by matching the drug to rare cancer types or cancers with specific mutations. The aim is to determine the efficacy of alectinib for ALK-positive rare cancers in adults, children, and adolescents/young adults as well as for common cancers in which ALK mutation or amplification is considered infrequent. STARTRK-NG is examining the use of entrectinib in children and young adults with recurrent or refractory solid tumors and primary central nervous system tumors harboring NTRK1/2/3, ROS1, or ALK aberrations [48]. The STARTRK-2 clinical trial is an open-label, multicenter, global phase 2 basket study of entrectinib for the treatment of patients with solid tumors harboring NTRK1/2/3, ROS1, or ALK gene rearrangements (fusions) [49]. These trials are demonstrating significant and durable responses in patients with tumors harboring specific mutations. The findings accumulated to date suggest that ALK fusion is not only an oncogenic protein, but also a therapeutic target in IMT. As of March 2023, ALK inhibitors have been covered by health insurance only for some non-small cell lung cancers and anaplastic large cell lymphoma in Japan. Combining ALK inhibitors with surgical treatment may improve the prognosis. In the future, ALK inhibitors may be a promising treatment option for unresectable IMT of the breast. More reports of patients with ALK-positive IMT are needed to determine this.

There are no well-established treatments for radiation therapy or chemotherapy; only surgical resection has good outcomes for IMT of the breast. Moreover, there are no definitive features with which to predict the risk of recurrence or metastasis after surgery. These facts emphasize the need for close follow-up, even if complete surgical resection is performed. Zhao et al. [25] reported that clinical physicians should perform regular follow-up after focal resection of IMT. Zhou et al. [23] recommended follow-up sonography every 6 months. Especially in children and younger adolescents, we suggest regular follow-up with ultrasonography instead of mammography. In short, the diagnosis and clinical follow-up can be critical for IMT management. Because no standard regimen has yet been established for IMT of the breast, a treatment plan based on accurate diagnosis must be made as early as possible, and regular follow-up is recommended to improve the survival of patients with this disease.

## Conclusion

We experienced a case of ALK-positive IMT of the breast in which we were able to achieve a preoperative diagnosis by pathological and immunohistochemical examination. ALK-positive IMT of the breast is extremely rare and shows nonspecific imaging findings despite having the characteristics of low-grade neoplasms with recurrence, invasion, and metastatic potential. Further investigations regarding the mechanism of tumor development, imaging findings for early detection, and the optimum treatment are essential for ALK-positive IMT. In addition, ALK inhibitors are expected to provide clinical benefit for ALK-positive IMT according to the preliminary results of several ongoing basket trials; therefore, we hope that combining ALK inhibitors with surgical treatment will improve patients’ clinical course. Our report emphasizes the importance of determining the treatment plan based on accurate examinations and regular follow-up after complete surgical resection to improve the prognosis of patients with primary ALK-positive IMT.

## Data Availability

The datasets supporting the conclusions of this article are included within the manuscript.

## Abbreviations

IMT: Inflammatory myofibroblastic tumor
ALK: Anaplastic lymphoma kinase
